# Modified Bilateral Perialar Crescent Flap for Reconstruction of Combined Upper Lip and Premaxillary Defect

**DOI:** 10.7759/cureus.5942

**Published:** 2019-10-18

**Authors:** Rinku George, Jyotsna Rajan, Mahathi Neralla, Santhosh P Kumar, Ahmed Elham Haque

**Affiliations:** 1 Oncology, Oral Cancer Institute, Saveetha Dental College, Chennai, IND; 2 Oral and Maxillofacial Surgery, Saveetha Dental College and Hospital, Chennai, IND

**Keywords:** oral squamous cell carcinoma, perialar crescent flap, lip reconstruction, local flaps

## Abstract

Many reconstruction methods are performed for combined defects of upper lip and premaxilla in oral cancer patients, which are complicated and multiple staged procedures, compromising the functional or structural unit. In this case report, we present a modification of the bilateral perialar crescent flap for reconstructing the combined defect of upper lip and premaxilla in a single stage. A patient diagnosed with well-differentiated squamous cell carcinoma of premaxilla and upper lip, involving a surgical defect of more than one-third but less than two-thirds of the lip underwent two cycles of neoadjuvant chemotherapy. Later wide local excision of the lesion and simultaneous reconstruction with a modified perialar crescent flap was performed in a single stage. Patient recovered uneventfully and tolerated the procedure well, without any complications. The patient was found to be satisfied with the functional and esthetic result. The reduced upper lip support which was a result of the bony defect of the premaxilla, was corrected with a dental prosthesis. This modification is a useful reconstruction tool for oral cancer patients with combined defects of upper lip and premaxilla.

## Introduction

Reconstruction of the upper lip and pre-maxillary segment is one of the greatest challenges for a surgeon. They both play a vital role in facial esthetics and oral function. The lip and its complex subunits make the reconstruction a very artistic and skillful procedure. If the tumor involves the pre-maxilla segment, upper gingivobuccal sulcus and is in direct continuity with the upper lip, then resection in the form of partial maxillectomy with extensive loss of upper lip tissue becomes mandatory. Full-thickness defects of one-third of the upper lip can be simply restored by direct apposition of the wound edges. When the defect is extensive and requires reconstruction of the lateral and medial small topographic subunits like philtrum, alar base, and nasolabial fold, a local or distant flap may be required [1]. Crescentic advancement is mainly used for reconstruction of alar base, perialar regions, and upper lip. In the cases of large defects of the upper lip, either unilateral or bilateral, this technique can be implemented [2]. First documented by Webster, the perialar crescentic advancement flap is the most widely used surgical technique for upper lip repair. The concept of this technique is similar to Burow’s triangle [3] in which a triangular tissue above and below the cheek pedicle is excised to facilitate the advancement of the flap without distortion [4]. Further, Webster modified the technique by excising bilateral crescentic cheek tissue and using the perialar incision for lengthening the upper skin edge of lip and cheek flap in repair of superficial defects, and for an approximation of this edge to the alae without any distortion in large tissue defects such as in full-thickness defects [3]. In this report, we present a case of oral squamous cell carcinoma of upper lip and premaxilla which was treated by wide local excision and reconstructed using the modified bilateral perialar crescent flap as a single-stage procedure.

## Case presentation

A 60-year-old female presented to the oral oncology outpatient department with painful ulceration in the upper lip, involving the gingivobuccal sulcus and premaxilla region of five months’ duration. Patient’s history revealed that she started using prosthesis for missing upper anterior teeth, after which the swelling started insidiously and progressed rapidly to attain the present size.

**Figure 1:**
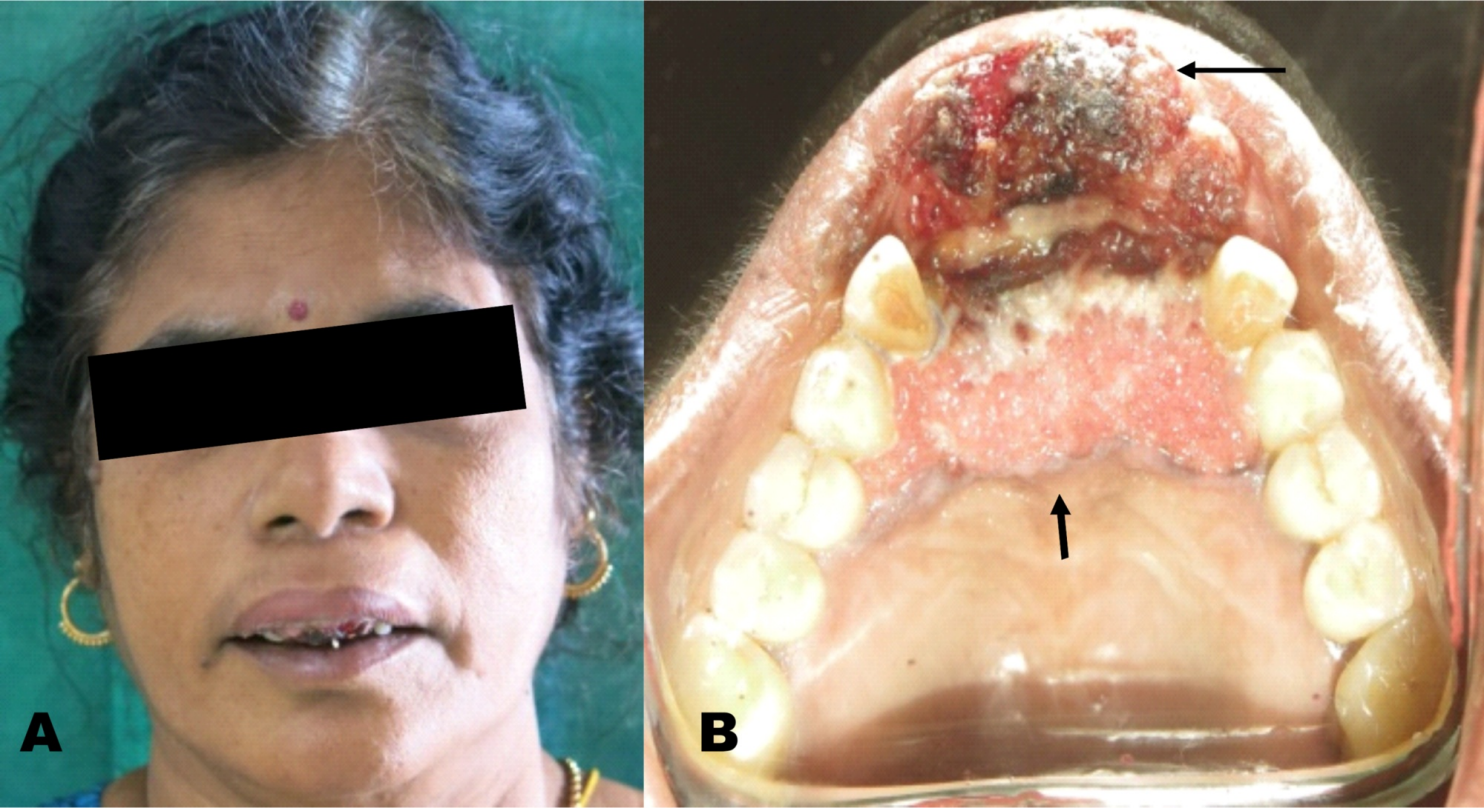
Extension of the lesion - (A) Extra-oral view (B) Intra-oral view

Clinical examination revealed a diffuse swelling in the upper lip, approximately 3 x 3 cm in maximum dimension, involving the alar base and philtrum. Intraoral examination revealed an ulcero-proliferative lesion crossing the midline; involving the anterior maxillary alveolus and gingivobuccal sulcus from 13 to 23 and traversing more than one-third of the upper lip (Figure 1). The growth was sessile and the edges of the lesion were irregular and ulcerated. The swelling was tender on palpation, with evident bleeding. The neck was palpated; however, no significant cervical nodes were found.

Magnetic resonance imaging of the head and neck revealed a mixed signal intensity lesion indicating destruction of the upper anterior alveolus, adjacent hard palate and soft tissue infiltration of gingivolabial sulcus and upper lip. Incisional biopsy was performed under local anesthesia which revealed well-differentiated squamous cell carcinoma. Chest radiograph revealed no significant findings. Based on the clinical signs, symptoms, the radiographic evidence, and the histopathological examination the lesion was diagnosed as squamous cell carcinoma of upper lip and premaxilla (clinical staging - T4a N0 M0, stage IV).

**Figure 2:**
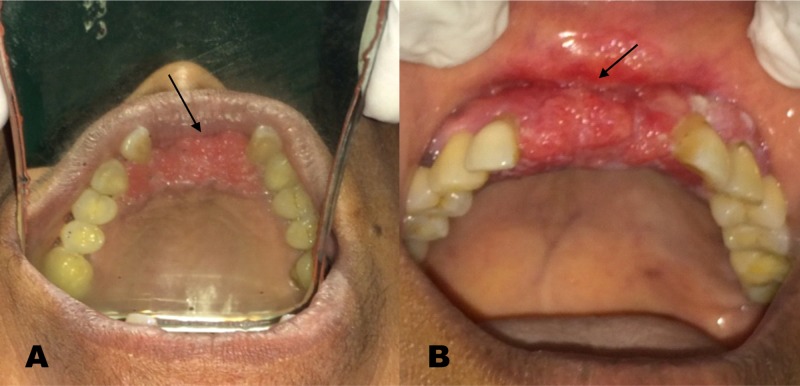
Lesion after completion of second neoadjuvant chemotherapy cycle - (A) Occlusal view (B) Frontal view

To reduce the size of the lesion and decrease the possibility of positive margins during resection, a medical oncologist’s opinion was sought, who suggested neo-adjuvant chemotherapy (NACT) with a combination of docetaxel, cisplatin, and 5-fluorouracil. Reassessment after three weeks following the first cycle of NACT showed a marked response with a considerable reduction in the size of the lesion. The second cycle of NACT was administered and on reassessment, the lesion had further reduced considerably (Figure 2).

**Figure 3:**
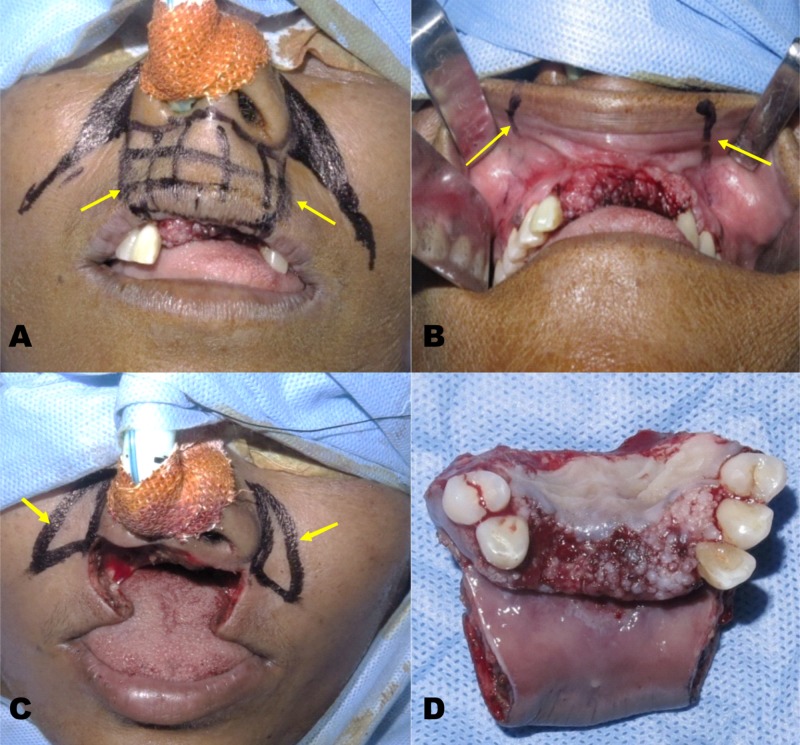
Resection of the lesion - (A) Extra-oral incision marking (B) Intra-oral incision marking (C) Wide local excision (D) Excised specimen

The surgical plan was wide local excision of two-thirds of upper lip with anterior maxillectomy and reconstruction with bilateral modified perialar crescent flap for the resulting surgical defect. This treatment plan was proposed considering the site and size of the lesion, and the feasibility to achieve adequate closure while reconstructing with the desired flap. Under general anesthesia, resection with 1 cm surgical margin for the lip lesion and 1.5 cm for premaxilla lesion was planned and markings made with indelible ink. Full thickness resection of the upper lip lesion and anterior maxillectomy including the first premolars bilaterally was performed. The excised specimen was sent for histopathological examination (Figure 3).

**Figure 4:**
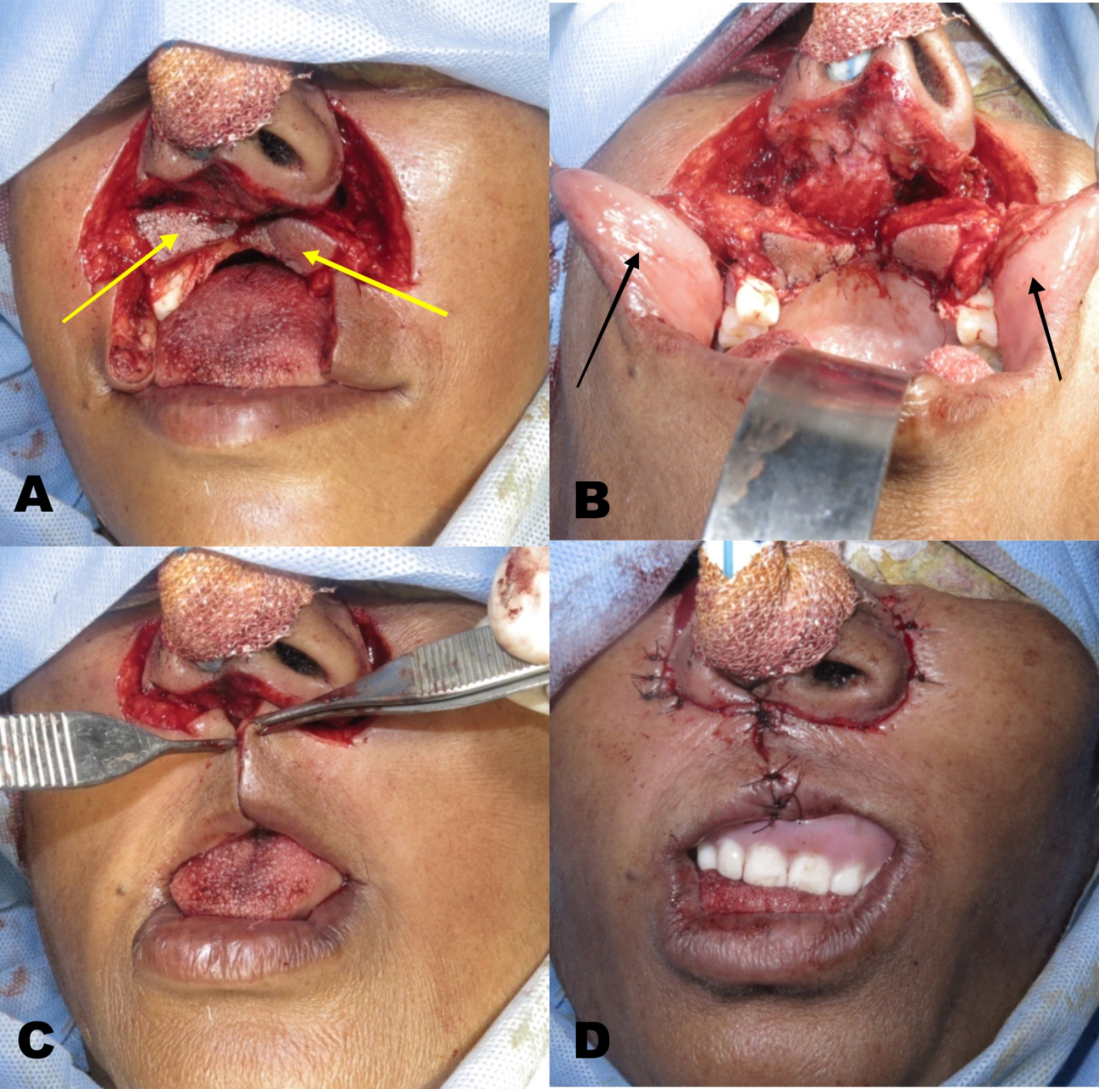
Reconstruction of the defect - (A) Modified bilateral perialar crescent flap (B) Full thickness cheek flap (C) Flap approximation (D) Final closure with temporary obturator

The medial and lateral vertical incision of the Burow’s triangle was placed along the nasolabial fold and the plane of dissection was kept below the underlying muscle. The crescentic flap margins for excision were kept wider and lower in this case, and the medial margin was extended around the alar base. The triangular portion of the Burow’s flap was rotated down intra-orally bilaterally, covering the surgical defect of the premaxilla region. Both the pedicled flaps were approximated cautiously and sutured together with the adjacent tissue margins. Buccal mucosa was freed and undermining of the cheek was done, therefore resulting in a full thickness cheek flap. Following this maneuver, the remnants of the lip were advanced to close the defect. The lip was sutured in layers and a temporary obturator was given for labial fullness (Figure 4). Due to the bony defect of premaxilla, there was a reduced upper lip support which was corrected with a removable dental prosthesis.

**Figure 5:**
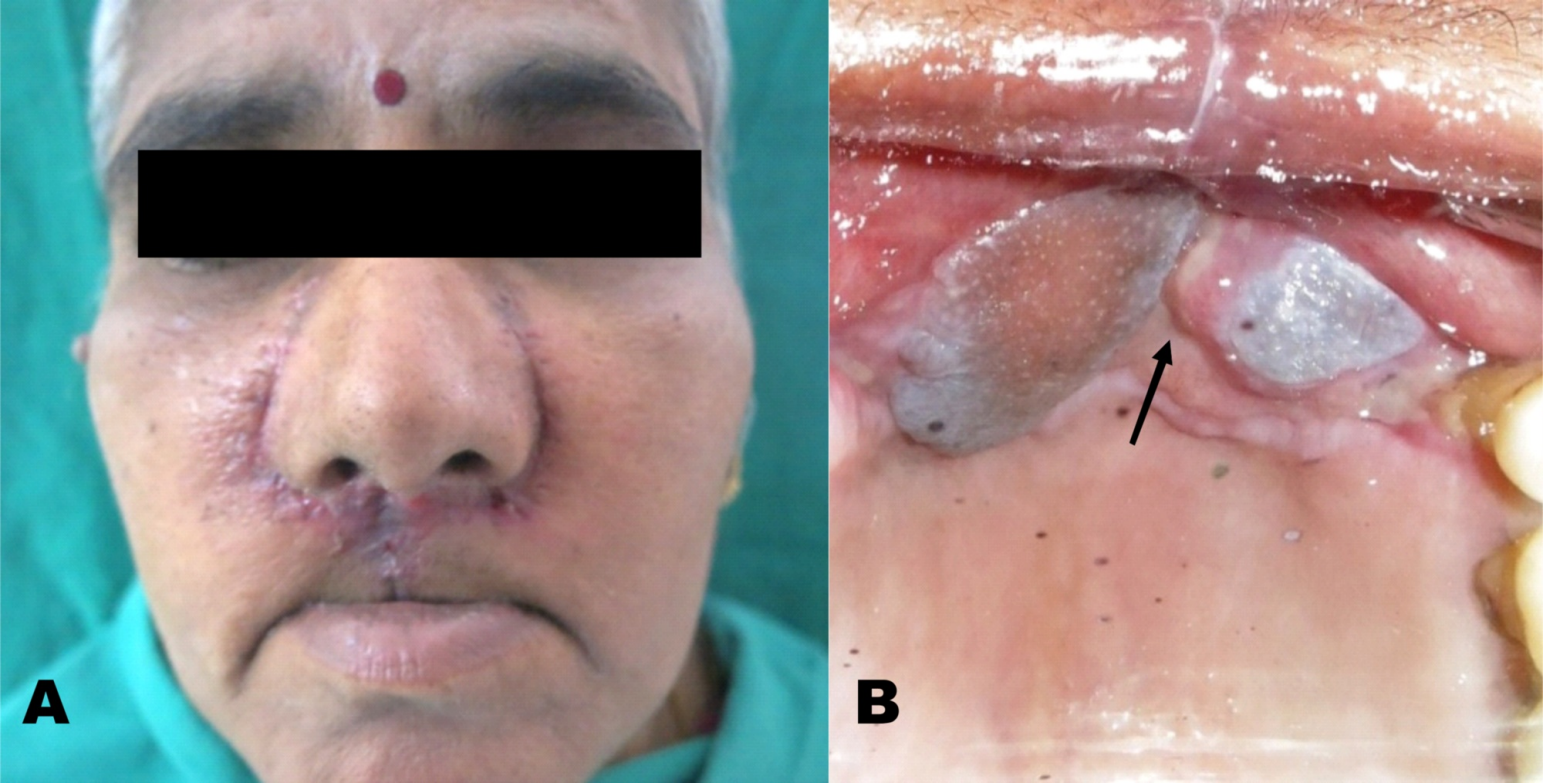
Post-operative assessment shows satisfactory wound healing - (A) Extra-oral view (B) Intra-oral view

Oral competence and speech were good and the patient was able to perform a wide range of facial expressions. One month postoperatively wound healing was satisfactory without oro-nasal communication or neurosensory complications (Figure 5).

## Discussion

There are various surgical modalities for correction of the combined defects of upper lip and premaxilla region which involve multi-staged procedure thereby compromising function and aesthetics. For combined defects of the premaxilla and upper lip, no single-stage procedure has been documented, as it requires both ablative and reconstructive surgical procedures for restoration of the defect. In our case, a modified bilateral perialar crescent flap was utilized for reconstructing the combined defect of premaxilla and full-thickness defect of the upper lip to achieve satisfactory results in a single stage.

In stage IV lesions, multimodality treatment should be favored, depending upon the site and extension of lesion [5]. Based on this, two cycles of NACT were planned to down-stage the tumor before surgery followed by adjuvant radiotherapy, to improve the local control and to prevent the loco-regional metastasis. Treatment with NACT proved to be beneficial in regressing the size of the lesion thereby favoring resection with negative margins followed by primary reconstruction.

**Figure 6:**
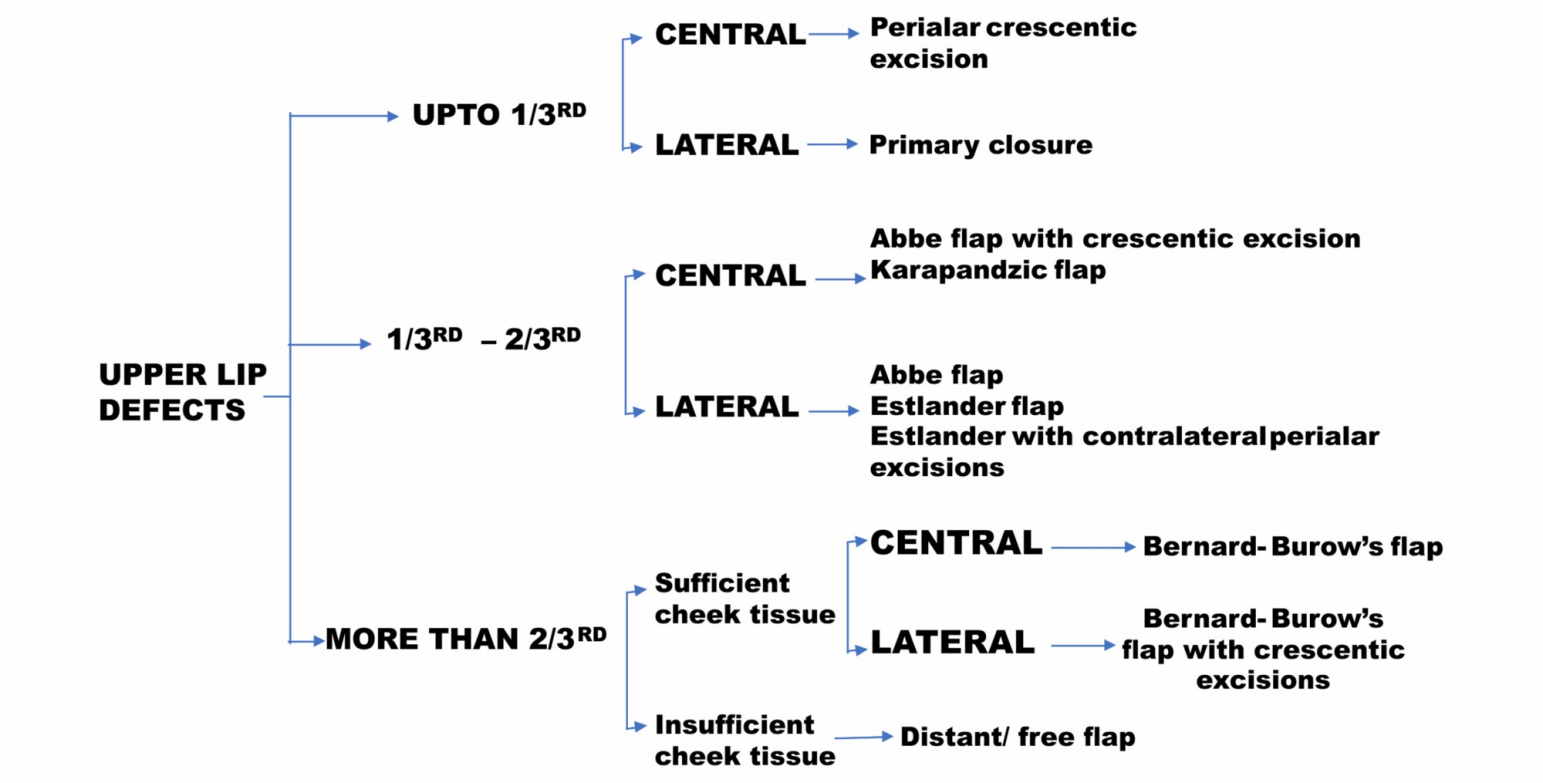
Protocol for reconstruction of upper lip defects

For the reconstruction of the upper lip defects, the reconstruction ladder starts from the simplest procedure and ascends in its complexity with emphasis on esthetics and conserving lip function. The aesthetic subunits of the upper lip reconstruction were elegantly described by Burget and Menick and are derived from the pertinent topography [6]. In the past 150 years, over 200 different reconstructive techniques ranging from typical V-W shaped excision to the creation of different types of flaps (used alone or in combination) have been described for lip reconstruction [7-8]. The choice of reconstruction depends upon the size and site of the upper lip defect (Figure 6) [9].

In our case, after wide local excision of the tumor, there was loss of premaxilla and portion of the upper lip. In the repair of full-thickness defects of the upper lip, bilateral perialar crescent flap enables better structural alignment of lip commissures and nasal unit with good functional results; but the loss of the premaxillary segment cannot be corrected in the same procedure [10]. Hence, we modified the bilateral perialar crescent flap by rotating it intraorally to reconstruct the premaxilla, which also prevented oro-nasal communication.

There were no complications associated with wound healing, adaptation, and vascularity of the flap. The advantages of this technique include reconstruction of combined upper lip and premaxilla in a single-stage thereby deferring the need for complicated secondary reconstructive procedures and preventing morbidity to the patient.

The limitation with the modified bilateral perialar crescentic advancement flap is microstomia [11]. The narrow mouth opening can be a significant problem for patients who wear dental prosthesis. Upper lip projection is dependent upon its underlying dentoalveolar segment [12]. In our case, there was loss of upper lip projection after partial maxillectomy resulting in poor esthetics. This was corrected with the help of a dental prosthesis.

## Conclusions

Modification of the bilateral perialar crescentic flap is a reliable technique that offers satisfactory functional and esthetic results. It is useful in reconstructing combined defects of upper lip and premaxilla in a one-stage surgical procedure, which reduces surgical cost and donor site morbidity.
